# Evolocumab Versus Statins and Placebo in Patients With Cardiovascular Disease and Comorbidities: A Systematic Review and Meta-Analysis

**DOI:** 10.7759/cureus.82037

**Published:** 2025-04-10

**Authors:** Jonathan A Casares, Arturo P Jaramillo, Sajidha Nizamudeen, Sanod Khan Abdul Samad

**Affiliations:** 1 Faculty of Medicine, Pontificia Universidad Católica del Ecuador, Quito, ECU; 2 General Practice, Universidad Estatal de Guayaquil, Machala, ECU; 3 Emergency Department, Travancore Medical College, Kollam, IND; 4 General Medicine, Bicol Christian College of Medicine, Legazpi, PHL

**Keywords:** cardiovascular education, cardiovascular effect, evolocumab, placebo controlled trials, statins

## Abstract

Evolocumab is a PCSK9 inhibitor designed to significantly reduce low-density lipoprotein cholesterol (LDL-C) levels. Unlike statins, which work by inhibiting 3-hydroxy-3-methylglutaryl coenzyme A reductase to reduce cholesterol synthesis in the liver, evolocumab enhances LDL receptor recycling, leading to more efficient clearance of LDL-C from the bloodstream. Compared to placebo, evolocumab shows a profound LDL-C lowering effect while also offering incremental benefit over statins, especially in high-risk patients or those with statin intolerance.

In this meta-analysis, the primary focus was on lipid-lowering efficacy and cardiovascular outcomes, making direct comparisons among evolocumab, statins, and placebo essential. Evolocumab consistently demonstrated a statistically significant reduction in LDL-C and, in several large-scale trials (such as Further Cardiovascular Outcomes Research With PCSK9 Inhibition in Subjects With Elevated Risk (FOURIER)), also showed a reduction in cardiovascular events, including myocardial infarction and stroke. It is important to specify that the most significant outcomes were both the substantial LDL-C reduction and the associated reduction in major adverse cardiovascular events (MACE).

For our meta-analysis, we generated three graphical models using Review Manager (RevMan) version 5.4 (Cochrane Collaboration, Copenhagen, Denmark) based on the selected studies. To conduct our systematic review, we extensively examined a total of 10 articles. The subgroup analyses in these studies looked at how well evolocumab worked on its own, with statins, and as an extra treatment for people who were already taking low or high doses of statins. Additionally, we compared the effectiveness of evolocumab vs. placebo in both individuals with and without cardiovascular conditions. Our findings indicated that evolocumab, whether used as monotherapy or alongside statins, demonstrated statistical significance (p = 0.01). Moreover, all reviewed studies reported statistically significant results (p < 0.05). According to our analysis, there is an urgent need for more research to build on this body of evidence and carry out larger randomized controlled trials (RCTs) to find the best timing and dose for each patient and avoid any possible long-term side effects.

## Introduction and background

When evaluating treatment efficacy, numerous long-term cardiovascular trials traditionally rely on survival analysis approaches that consider only the first cardiovascular event experienced by a patient during the study period. Such methods, while common, may not fully capture the comprehensive clinical impact of a therapy, especially when the primary outcome measured is composed of various distinct cardiovascular events. Relying solely on first-event analysis can limit the understanding of a treatment's true effectiveness because participants experiencing nonfatal events typically continue to be monitored, potentially undergoing additional cardiovascular episodes. Low-density lipoprotein cholesterol (LDL-C)-lowering trials have acknowledged this limitation, prompting researchers to examine both initial and subsequent cardiovascular events when comparing high-intensity to moderate-intensity statins [1]. For instance, in the Pravastatin or Atorvastatin Evaluation and Infection Therapy-Thrombolysis in Myocardial Infarction (PROVE IT-TIMI) trial and the Incremental Decrease in Endpoints Through Aggressive Lipid Lowering (IDEAL) trial, results demonstrated that achieving lower LDL-C levels with high-intensity statins effectively reduced both initial cardiovascular events and the total event count relative to moderate-intensity statin treatments [2,3]. Furthermore, the Improved Reduction of Outcomes: Vytorin Efficacy International Trial (IMPROVE-IT) established that additional LDL-C reductions obtained by supplementing statin therapy with the non-statin lipid-lowering medication ezetimibe significantly decreased both initial and recurrent cardiovascular events compared to treatment using simvastatin alone [4,5]. Despite continuous statin therapy, individuals with stable cardiovascular disease (CVD) persistently face substantial risk for recurrent cardiovascular events. Evolocumab, a PCSK9 inhibitor, has been notably effective in reducing the likelihood of first cardiovascular events among patients receiving statins for atherosclerotic cardiovascular disease (ASCVD) [6,7]. Further Cardiovascular Outcomes Research With PCSK9 Inhibition in Subjects With Elevated Risk (FOURIER) trials also evaluated evolocumab's efficacy in diminishing the total cardiovascular event burden among statin-treated individuals with stable vascular disease [8,9].

A notable example of high cardiovascular morbidity and mortality is observed in China, which bears a substantial global burden of diabetes, with a prevalence rate estimated at approximately 10% as of 2013 [2]. Diabetes is widely recognized as a significant CVD risk factor among Chinese populations, mirroring observations from Western countries [10]. Globally, and in China specifically, patients diagnosed with type 2 diabetes mellitus (T2DM) exhibit notably increased risks of severe cardiovascular complications such as cardiovascular mortality, myocardial infarction, hypertension, and stroke [11]. Given that elevated LDL-C is directly correlated with heightened CVD risks, LDL-C management remains a primary treatment objective for dyslipidemia in T2DM patients. Chinese treatment guidelines advocate the use of statins to maintain LDL-C below 1.9 mmol/L for these high-risk groups. Nevertheless, despite statin availability, a substantial portion of Chinese T2DM patients (approximately 61-73%) treated in hospitals or community healthcare facilities fail to reach recommended LDL-C targets [12,13]. Statin intolerance, characterized by adverse reactions, appears more frequently among Chinese individuals compared to global populations, suggesting that poor LDL-C control in these patients may stem from difficulties in tolerating high-intensity statin regimens [13]. Consequently, novel lipid-lowering therapies are urgently needed to complement maximally tolerated statin doses, addressing the LDL-C treatment gaps observed in China.

Phase 2 and 3 clinical studies have consistently demonstrated evolocumab’s effectiveness, a human monoclonal antibody targeting PCSK9, in significantly reducing LDL-C without negatively influencing glycemic control metrics, regardless of diabetes status [13]. The global, double-blind, randomized phase 3 Evolocumab Efficacy for LDL-C Reduction in Subjects With T2DM Background Statin (BERSON) trial (N=986), with substantial Chinese participation, evaluated evolocumab's LDL-C lowering effects in combination with atorvastatin in T2DM patients diagnosed with hyperlipidemia or mixed dyslipidemia. The global findings indicated significant LDL-C reductions and improvements in other lipid profiles with evolocumab compared to placebo. Additionally, evolocumab exhibited strong safety and tolerability profiles, with no adverse glycemic control effects. A pre-specified subgroup analysis from the BERSON trial specifically assessed evolocumab's efficacy and safety in the Chinese patient subgroup, reinforcing its therapeutic potential [13].

Reducing LDL-C via statins significantly mitigates cardiovascular risks in atherosclerotic patients. Even greater benefits have been observed with intensive statin therapy, either alone or combined with ezetimibe and/or PCSK9 inhibitors [13]. Treatment guidelines, therefore, strongly advocate achieving very low LDL-C levels for high-risk cardiovascular patients. Despite these recommendations, fewer than half of patients with established ASCVD receive sufficiently aggressive lipid-lowering treatments to achieve recommended LDL-C targets [11]. Patients with recent acute coronary syndrome (ACS) represent a particularly high-risk group [10,11]. Acute coronary events frequently result from ruptured atherosclerotic plaques enriched with lipids and inflammatory cells, typically characterized by a thin fibrous cap less than 65 micrometers in thickness. The presence of similar plaques elsewhere in the coronary vasculature underscores the systemic nature of atherosclerosis and emphasizes the need for aggressive, comprehensive risk factor management post-ACS [4-6].

Adding PCSK9 inhibitors to statins is notably beneficial in patients experiencing recent myocardial infarctions with residual multivessel coronary disease, likely due to plaque stabilization, particularly in vulnerable thin-cap fibroatheroma. Clinical evidence demonstrates that coronary atherosclerosis regression is directly proportional to LDL-C reduction achieved via intensive statin monotherapy or combined therapy involving ezetimibe and PCSK9 inhibitors [10]. Optical coherence tomography (OCT), a high-resolution imaging technology, allows detailed characterization of plaque structures and fibrous caps. Although statins have shown the potential to thicken plaque fibrous caps, the impact of PCSK9 inhibitors on plaque morphology assessed via serial OCT has not yet been conclusively explored [8]. The High-Resolution Assessment of Coronary Plaques in a Global Evolocumab Randomized Study (HUYGENS) trial was specifically designed to assess whether PCSK9 inhibitor addition to high-intensity statin regimens positively influences coronary plaque morphology [6].

This systematic review and meta-analysis aimed to comprehensively evaluate recent research comparing evolocumab, statins, and placebo interventions in treating patients with CVD and related comorbidities. By critically assessing available evidence, we sought to enhance clinical knowledge and optimize treatment strategies to improve patient outcomes.

## Review

Methods

Review Record and Search for Studies

In conducting this systematic review, we adhered to the protocols specified in the Preferred Reporting Items for Systematic Reviews and Meta-Analyses (PRISMA) [14]. Independent researchers conducted the selection of articles through thorough searches in databases like PubMed, PubMed Central (PMC), and Cochrane Library. Table 1 provides a detailed overview of the specific search strategies employed. 

**Table 1:** Search strategy for databases

Search strategy	Databases used	Number of papers identified
Evolocumab AND Cardiovascular comorbidities AND Statins AND Placebo vs Evolocumab	Pubmed	1284
(Evolocumab [Title/Abstract]). (Statins [Title/Abstract]). ((Cardiovascular comorbidities [Title/Abstract]); OR (Cardiovascular conditions [Title/Abstract]); AND ((Statins treatment [Title/Abstract]); OR (Placebo vs Evolocumab [Title/Abstract])	Pubmed Central (PMC)	4464
"Evolocumab [tw]" AND "Statins [tiab]" AND "Cardiovascular comorbidities [all]"	Cochrane Library	522

Inclusion and Exclusion Criteria

Two distinct authors utilized the Covidence software (Veritas Health Innovation, Melbourne, Australia) to examine the search results obtained from three databases based on established inclusion and exclusion criteria, as outlined in Table 2. 

**Table 2:** Inclusion and exclusion criteria

Inclusion	Exclusion
Free, full text about the related subject	Articles that include pharmaceutical trials
Articles from the past 10 years	Articles from 2015 and before
English-language articles	Non-English studies
Prospective or retrospective studies	Case reports, systematic reviews, or literature reviews
Human trials	Animal trials

Data Collection Process

To ensure consistency and minimize bias, we utilized a standardized data extraction form that was developed a priori and pilot-tested on a subset of eligible studies. This form was designed to capture key study-level and outcome-level information, including:

(i) Study characteristics: author, year of publication, study design, country, sample size, funding source.

(ii) Patient characteristics: age, sex distribution, baseline LDL-C levels, presence of comorbidities (e.g., diabetes, ASCVD), and statin tolerance/intolerance status.

(iii) Intervention details: type, dosage, and duration of evolocumab and comparator treatments (e.g., statins, placebo).

(iv) Outcomes extracted: LDL-C reduction, major adverse cardiovascular events (MACE), all-cause mortality, adverse effects, and follow-up duration.

Data extraction was conducted independently by two reviewers, and any discrepancies were resolved through discussion and consensus. If consensus could not be reached, a third reviewer adjudicated the final decision. This approach helped ensure accuracy, reduce individual bias, and enhance the reproducibility of our findings.

Risk of Bias Evaluation

We utilized the Cochrane Risk of Bias (RoB) tool (Cochrane Collaboration, London, UK) to evaluate the integrity and reliability of the chosen studies, as it is a well-established standard for assessing randomized controlled trials (RCTs). This tool is highly regarded for its ability to assess the quality of RCTs [15]. Our team performed a meticulous and impartial evaluation of every study, addressing any differences of opinion among the reviewers through in-depth discussions, which guaranteed a complete and equitable analysis of possible biases.

Statistical Methodology

For all statistical analyses, we employed Review Manager (RevMan) version 5.4 (2020), created by the Cochrane Collaboration at the Nordic Cochrane Centre in Copenhagen, Denmark. The results of the studies were expressed through mean differences, along with 95% confidence intervals (CI). An odds ratio (OR) effects model was utilized for the aggregation of data from various studies. In cases where studies did not provide standard deviations or standard errors, we utilized the statistical methods described by Mantel-Haenszel [16]. Considering the diverse designs and demographic features of the studies included, a fixed-effect model was chosen instead of a random-effect model because it is better suited for handling the anticipated high variance. Forest plots were essential for visually summarizing the aggregated results, and the chi-square test was key in identifying any differences among subgroups. The degree of variability among the studies was measured using the Higgins I^2^ statistic. Funnel plots were examined visually to evaluate publication bias, using a significance threshold of p < 0.05.

Research Outcomes and Study Selection

**Figure 1:**
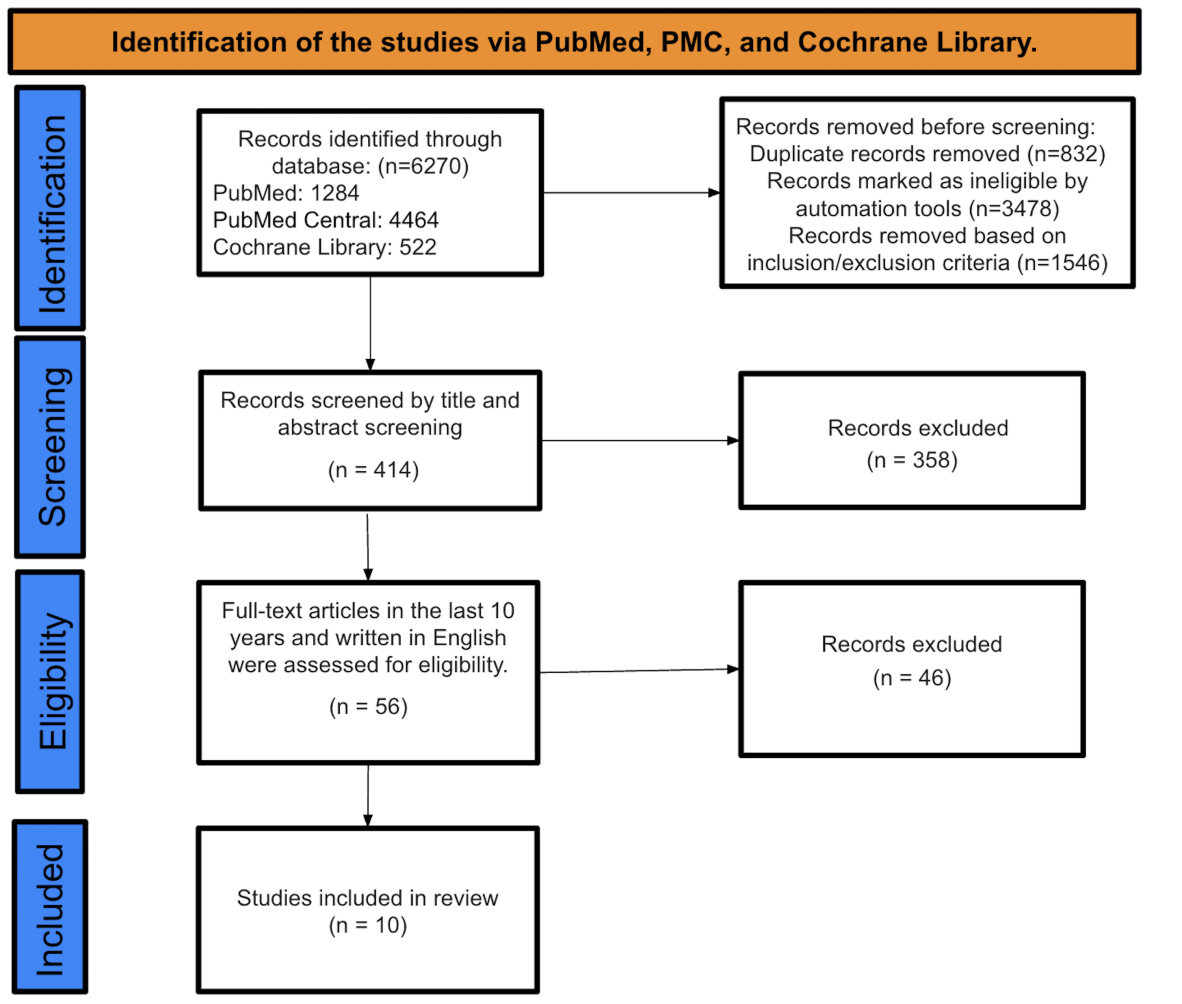
PRISMA flow diagram of the search and review process PRISMA: Preferred Reporting Items for Systematic Reviews and Meta-Analyses

We conducted an initial search through various databases such as PubMed (example of it: Stone et al. article) [17], Cochrane Library, and PMC, which resulted in a total of 6,270 studies. Following the application of inclusion and exclusion criteria, 1,546 studies were found to be ineligible, while automation tools flagged an extra 3,478 articles as irrelevant. During our review process, we removed 832 duplicates. After reviewing the titles and abstracts of the 414 articles, we excluded 358 studies that did not fit our research objectives. Following a thorough review considering the accessibility of complete texts in English and their publication within the past 10 years, 46 of the 56 studies that were initially screened were eliminated. In the end, just 10 studies fulfilled all the criteria and were incorporated into our final data analysis, as shown in Figure 1, which demonstrates the process of identifying and selecting studies through different databases and registers. 

Table 3 shows an in-depth description of the articles that were finally decided to be used.

**Table 3:** Details of the articles selected for review RCT: Randomized controlled trial; Q2W: Every two weeks; QM: Monthly; LDL-C: Low-density lipoprotein cholesterol; MetS: Metabolic syndrome; NSTEMI: Non-ST-segment elevation myocardial infarction; PEP: Primary endpoint; RR: Incidence rate ratio; HR: Hazards ratio; FOURIER: Further Cardiovascular Outcomes Research With PCSK9 Inhibition in Subjects With Elevated Risk

Author	Year of publication	Study design	Primary research	Outcome evaluation
Murphy et al. [1]	2019	RCT	The research involved 27,564 patients diagnosed with stable atherosclerotic disease who were undergoing statin treatment. The data analysis took place from May 2017 to February 2019.	Evolocumab demonstrated an 18% reduction in the overall incidence of PEP events (RR, 0.82; 95% CI, 0.75-0.90; P < .001). This reduction included both initial events (HR, 0.85; 95% CI, 0.79-0.92; P < .001) and subsequent occurrences (RR, 0.74; 95% CI, 0.65-0.85).
Chen et al. [13]	2018	RCT	Patients began with a baseline treatment of atorvastatin at a dose of 20 mg per day. Following this initial period, they were randomly assigned in a 2:2:1:1 ratio to receive either evolocumab 140 mg Q2W, evolocumab 420 mg QM, or placebo administered either every two weeks or monthly.	Compared to placebo, evolocumab significantly decreased LDL-C levels at week 12 (Q2W, -85.0%; QM, -74.8%) as well as at the averaged measurements from weeks 10 and 12 (Q2W, -80.4%; QM, -81.0%).
Nissen et al. [18]	2016	RCT	Patients qualified for the study if they had LDL-C concentrations higher than the target levels outlined by the National Cholesterol Education Project Adult Treatment Panel III guidelines, corresponding to their specific coronary heart disease risk category, and had a documented intolerance to a minimum of three statins or at least two statins.	Outcomes assessed included changes from baseline in LDL-C, the proportion of patients achieving LDL-C levels below 70 mg/dL, and percent changes from baseline in total cholesterol, non-high-density lipoprotein cholesterol, and apolipoprotein B.
Giugliano et al. [19]	2016	RCT	Two subgroups of patients diagnosed with stable atherosclerotic cardiovascular disease who were already on statin therapy were analyzed.	Among the 7,533 patients treated with maximal-potency statins, evolocumab significantly lowered the risk of the primary endpoint (HR, 0.86; 95% CI, 0.75-0.98). This benefit was comparable to the effect observed in the 20,031 patients who were not on maximal-potency statins (P = 0.88 for interaction).
Nicholls et al. [20]	2016	RCT	Participants diagnosed with angiographic coronary disease were randomly assigned to receive monthly subcutaneous injections of either evolocumab or placebo for 76 weeks, alongside ongoing statin therapy.	Compared to the placebo group, patients receiving evolocumab experienced significantly lower mean time-weighted LDL-C levels (RR, 0.74; 95% CI, 0.65-0.85; P < .001).
Sabatine et al. [21]	2017	RCT	The study enrolled 27,564 patients diagnosed with atherosclerotic cardiovascular disease who had LDL-C levels of at least 70 mg/dL and were undergoing statin therapy. Participants were randomly assigned to receive either evolocumab or a corresponding placebo, administered via subcutaneous injections.	The addition of evolocumab to statin therapy reduced LDL-C to a median level of 30 mg and was associated with a decreased incidence of cardiovascular events. These results indicated that individuals with atherosclerotic cardiovascular disease gain significant benefits from achieving lower LDL-C levels than those currently recommended by standard treatment goals.
Nicholls et al. [22]	2018	RCT	In total, 968 patients with coronary artery disease receiving statin therapy underwent serial coronary intravascular ultrasound imaging at baseline and after 76 weeks of monthly treatment with either evolocumab or placebo.	When compared to statin therapy alone, evolocumab achieved greater reductions in LDL cholesterol and promoted significant regression in percent atheroma volume as well as total atheroma volume.
Koba et al. [23]	2020	RCT	This RCT was done within 12 weeks. A total of 61 patients were randomly assigned to receive either evolocumab (40 patients) or ezetimibe (21 patients). Evolocumab significantly lowered LDL-C levels compared to ezetimibe, with reductions of approximately 39-40% at weeks 10 and 12. The differences were statistically significant, with confidence intervals confirming a strong effect (adjusted p < 0.0001). Eligible patients were randomly assigned in a 2:2:1:1 ratio into four treatment arms: evolocumab 420 mg every four weeks plus daily oral placebo, evolocumab 140 mg every two weeks, or control groups.	A total of 61 patients were randomized to either evolocumab or ezetimibe treatment. The difference in LDL cholesterol reduction between the evolocumab and ezetimibe groups was -39.4%.
Deedwania et al. [24]	2021	RCT	Data collection occurred from February 2013 through November 2016. The FOURIER randomized controlled trial enrolled patients globally who had stable atherosclerotic cardiovascular disease and were receiving statin therapy; participants were randomly assigned to receive evolocumab or placebo and were followed for a median duration of 2.2 years.	Evolocumab significantly decreased LDL-C levels in patients both with and without MetS.
Nicholls et al. [25]	2022	RCT	Patients were randomized in a 1:1 ratio using a block size of four, with stratification based on whether statin therapy had been administered for more than four weeks before screening. Randomization was conducted via an interactive voice response system, assigning patients to monthly subcutaneous injections of either evolocumab or placebo for a duration of 52 weeks.	The early introduction of evolocumab alongside intensive statin therapy in patients presenting with NSTEMI demonstrated additional improvements in plaque characteristics.

Table 4 shows the Cochrane RoB tool used for the included RCTs.

**Table 4:** Bias assessment of the included RCTs using Cochrane RoB tool +: Article does not have any of the biases described in the table; -: Article bias is present; ?: Article bias is not found in the study. RoB: Risk of Bias; RCTs: Randomized controlled trials

Studies	Random sequence generation (selection bias)	Allocation concealment (selection bias)	Blinding of participants	Blinding of personnel/care providers (performance bias)	Blinding of outcome assessor (detection bias)	Incomplete outcome data (attrition bias)	Selective reporting (reporting bias)	Other biases	Overall
Murphy et al. [1]	+	+	+	+	+	+	+	-	7/8
Chen et al. [13]	+	+	+	+	-	+	+	-	7/8
Nissen et al. [18]	+	+	+	+	?	+	+	-	7/8
Giugliano et al. [19]	+	+	+	+	+	+	+	-	7/8
Nicholls et al. [20]	+	+	+	+	+	+	+	-	7/8
Sabatine et al. [21]	+	+	+	+	?	+	+	-	6/8
Nicholls et al. [22]	+	+	+	+	+	+	+	-	7/8
Koba et al. [23]	+	+	+	+	?	+	+	-	6/8
Deedwania et al. [24]	+	+	+	+	?	+	+	-	6/8
Nicholls et al. [25]	+	+	+	+	?	+	+	-	6/8

Meta-analysis of outcomes

**Figure 2:**
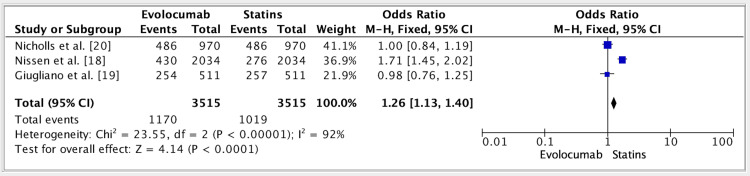
Forest plot for studies about the efficacy of evolocumab vs. statins groups References: [18-20]

The results of three studies showed an OR of 1.26 (fixed effect, 95%) for the efficacy of evolocumab vs. statins groups. CI was 1.13-1.40, P value was 0.01, and heterogeneity (I^2^) was 92% (Figure 2).

**Figure 3:**
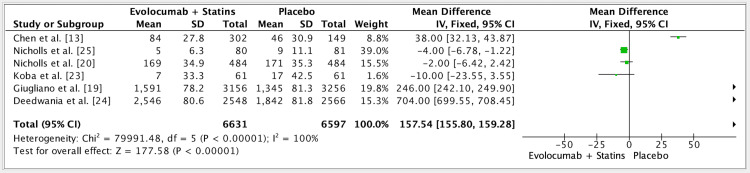
Forest plot for studies about the efficacy of evolocumab + statins vs. placebo groups References: [13,19,20,23-25]

The results of six studies showed a mean difference of 157.54 (fixed effect, 95%) in the efficacy of evolocumab + statins vs. placebo groups. CI was 155.80-159.28, P value was 0.01, and I^2^ was 100% (Figure 3).

**Figure 4:**
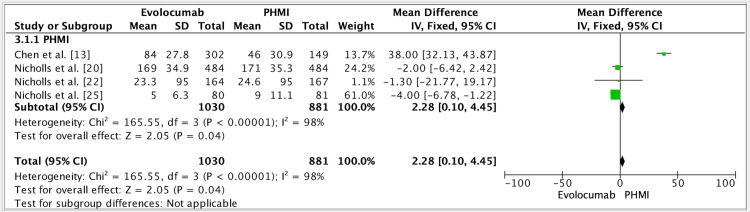
Forest plot for studies about the efficacy of evolocumab in patients with PHMI References: [13,20,22,25] PHMI: Past medical history of myocardial infarction

The results of four studies showed a mean difference of 2.28 (fixed effect, 95%) in the efficacy of evolocumab in patients with a past medical history of myocardial infarction (PHMI). CI was 0.10-4.45, P value was 0.01, and I^2^ was 98% (Figure 4).

**Figure 5:**
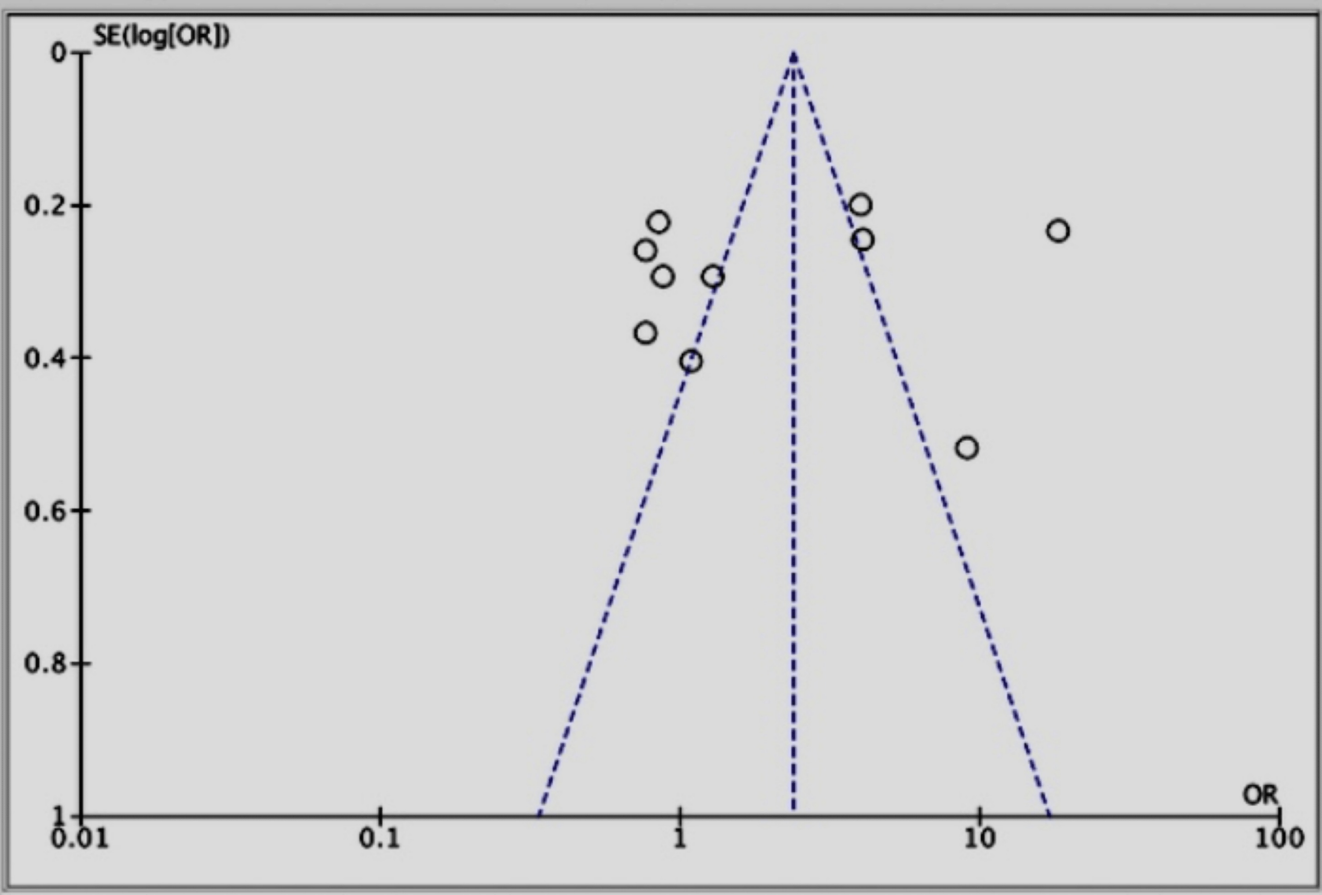
Funnel plot for all included studies about the efficacy of evolocumab + statins vs. placebo groups References: [1,13,18-25]

Publication bias was seen in all of the studies (Figure 5).

Discussion

Monoclonal antibodies targeting PCSK9 have emerged as a widely recognized and effective treatment option for reducing LDL-C levels and consequently lowering the risk of major cardiovascular events. Evolocumab, specifically, is a fully human monoclonal antibody that binds to PCSK9, resulting in significant reductions in LDL-C (approximately 60% at the conclusion of the dosing period and between 65% and 68% on average throughout the treatment interval). In this context, our systematic review analyzed 10 studies examining the efficacy of evolocumab, specifically comparing it to placebo and statin therapies in patients presenting with CVD and related comorbidities. 

Our findings should be interpreted in light of several important considerations. While the Global Assessment of Plaque Regression With a PCSK9 Antibody as Measured by Intravascular Ultrasound (GLAGOV) trial demonstrated modest plaque regression with evolocumab, Nicholls et al. (2018) did not observe significant changes in plaque composition [19,24]. These differences may be attributed to variations in imaging methods, patient populations, follow-up duration, and study design. Furthermore, the included studies encompassed heterogeneous populations, including statin-intolerant patients and individuals with established ASCVD, limiting the generalizability of results. Subgroup findings, such as greater LDL-C reductions in patients with higher baseline levels or enhanced cardiovascular benefits in very high-risk groups, highlight the need for more stratified analyses in future research [21].

Although LDL-C lowering is the primary mechanism of action, the observed cardiovascular benefits may also involve pleiotropic effects of PCSK9 inhibitors, including potential anti-inflammatory actions and plaque stabilization. However, these remain incompletely understood [21]. The cost of evolocumab remains a major barrier to widespread use, and current clinical guidelines recommend it primarily for statin-intolerant or very high-risk patients rather than routine use in all individuals with hyperlipidemia. While the safety profile appears favorable with no evidence of glycemic worsening, long-term data on neurocognitive effects, immunogenicity, and adherence are limited and require further investigation.

High heterogeneity across studies may stem from differences in endpoints (e.g., first vs. total events), patient characteristics, and treatment regimens. Although we did not conduct subgroup or meta-regression analyses due to limited data and inconsistent reporting, we acknowledge this as a limitation. Similarly, while publication bias was assessed using the funnel plot, we did not apply the trim-and-fill method due to concerns about its reliability under high heterogeneity and small sample sizes. Finally, ongoing and future studies should explore the long-term cardiovascular benefits of PCSK9 inhibitors beyond LDL-C reduction and evaluate combination therapies (e.g., with inclisiran or anti-inflammatory agents) to address residual cardiovascular risk in high-risk populations [19].

Murphy et al.'s RCT demonstrated that incorporating evolocumab into ongoing statin treatment yielded significant clinical improvements, notably reducing overall primary endpoint occurrences. These reductions were largely attributable to decreases in myocardial infarction, stroke, and the need for coronary revascularization procedures. Compared with placebo, the inclusion of evolocumab prevented more than twice as many cardiovascular events, particularly when evaluating total events rather than solely first occurrences. Such findings reinforce the importance and benefits of persistently aggressive lipid-lowering strategies to minimize recurrent cardiovascular episodes [1].

The 2016 RCT by Nicholls et al. observed that, despite reaching markedly low LDL-C levels through evolocumab therapy, only about two-thirds of the patients experienced regression of atherosclerotic plaques. However, the GLAGOV trial assessments were conducted after 18 months, shorter than the 24-month durations utilized in comparable high-intensity statin therapy studies. Thus, it remains plausible that prolonged evolocumab therapy could have produced regression in a larger patient proportion [20]. Nevertheless, the GLAGOV trial points to inherent biological constraints potentially limiting the percentage of individuals who experience regression even with highly effective LDL-C lowering, suggesting that additional factors beyond LDL-C influence disease progression. Future investigations into these contributing elements could uncover additional therapeutic targets [20].

Nissen et al.'s Goal Achievement After Utilizing an Anti-PCSK9 Antibody in Statin-Intolerant Subjects (GAUSS)-3 RCT focused on patients who met strict criteria for statin intolerance, a group with significant clinical unmet needs. This patient population exhibited extremely high baseline LDL-C values, approximately 200 mg/dL (levels deemed highly unacceptable even in primary prevention scenarios). Nearly half the patients recruited had existing coronary, cerebrovascular, or peripheral artery disease, and over 50% fell within the highest risk category as defined by the National Cholesterol Education Project Adult Treatment Panel III guidelines. The necessity for substantial LDL-C reduction in these individuals was clearly evident [17,18]. The GAUSS-3 outcomes aim to inform clinicians regarding the comparative effectiveness of evolocumab vs. ezetimibe and contribute to regulatory understanding about the potential therapeutic role of PCSK9 inhibitors in managing patients facing significant treatment challenges [17,18].

Giugliano et al.'s RCT highlighted evolocumab’s ability to safely reduce cardiovascular events in patients with stable ASCVD. Importantly, these benefits persisted regardless of baseline LDL-C levels being below or above 70 mg/dL and irrespective of whether patients were on maximal or submaximal intensity statin regimens [18]. These results endorse the use of evolocumab beyond current guideline recommendations, advocating for LDL-C reductions to approximately 20 mg/dL, even in high-risk patients starting below standard treatment thresholds. Furthermore, Sabatine et al. reported that the addition of evolocumab to statin treatment lowered LDL-C levels by an average of 59% relative to placebo, without evidence of diminishing efficacy over time. Their cardiovascular outcomes study demonstrated that the addition of evolocumab significantly reduced cardiovascular risk by 15% for the primary composite endpoint, including cardiovascular death, myocardial infarction, stroke, hospitalization for unstable angina, or coronary revascularization, and achieved a more substantial 20% risk reduction for the clinically critical secondary endpoint of cardiovascular death, myocardial infarction, or stroke [21]. Thus, evolocumab effectively lowers LDL cholesterol levels to a median of approximately 30 mg/dL and significantly diminishes cardiovascular risks, highlighting that patients with ASCVD benefit from lowering LDL-C below current treatment targets [21].

The 2018 study by Nicholls et al. indicated that the addition of evolocumab to existing statin therapy did not significantly alter plaque composition compared to statin monotherapy alone, suggesting limited additional benefit from assessing plaque morphology via virtual histology imaging beyond basic plaque burden measurements [25]. Conversely, research by Koba et al. demonstrated superior LDL-C reduction with evolocumab compared to ezetimibe in Japanese patients with hypercholesterolemia and statin intolerance, suggesting a favorable balance between efficacy and safety [23]. These findings align closely with results obtained in non-Japanese cohorts within the GAUSS-2 trial.

Deedwania et al. concluded that individuals with ASCVD and metabolic syndrome (MetS) continue facing elevated cardiovascular event risks despite receiving statin therapy [24]. Evolocumab treatment substantially reduced major cardiovascular events in ASCVD patients with MetS, independent of diabetes status. Given the higher baseline risk in patients with diabetes and MetS, the absolute risk reduction observed with evolocumab was notably greater in these groups compared to those without these conditions [24]. Importantly, evolocumab did not exacerbate glycemic control nor increase diabetes incidence, further confirming its safety and efficacy. These insights can guide clinical decision-making regarding appropriate patient selection for PCSK9 inhibitor treatment [24].

Finally, Nicholls et al.’s study highlighted that initiating evolocumab early in conjunction with intensive statin therapy among patients experiencing non-ST-segment elevation myocardial infarction (NSTEMI) provided incremental improvements in plaque characteristics. The data indicate the potential stabilization of vulnerable plaques following an ACS, reinforcing the importance of promptly implementing aggressive lipid-lowering therapies post-ACS to mitigate future cardiovascular risks [25].

Collectively, this systematic review synthesized various perspectives from the selected articles, offering a comprehensive understanding of current clinical evidence surrounding evolocumab therapy. This consolidated evidence base would assist healthcare providers in making informed decisions regarding evolocumab, statins, or their combination for managing patients with CVD and associated comorbidities.

Limitations

The studies analysed in this meta-analysis vary considerably in sample size, ranging from smaller trials with limited participants to large-scale multicenter investigations. This variability introduces challenges in terms of generalisability, as findings from smaller studies may not fully represent broader populations. Furthermore, discrepancies in study design, duration of follow-up, and participant demographics may have influenced the outcomes reported. While large trials provide robust data, smaller studies contribute valuable insights but require careful interpretation. Addressing these limitations through future research can help refine the clinical applicability of evolocumab treatment.

## Conclusions

In conclusion, evolocumab effectively reduces LDL-C levels and significantly lowers the risk of cardiovascular events in patients with CVD and comorbidities. This review highlights its benefits in combination with statins or for statin-intolerant patients, noting improvements in myocardial infarction, stroke, and coronary revascularization rates. Although not all patients experience plaque regression, prolonged therapy and exploration of additional factors beyond LDL-C could enhance outcomes. Evolocumab demonstrates consistent safety and effectiveness across diverse patient groups, supporting its integration into aggressive lipid-lowering strategies to improve cardiovascular care.
